# Using a Body-Fixed Sensor to Identify Subclinical Gait Difficulties in Older Adults with IADL Disability: Maximizing the Output of the Timed Up and Go

**DOI:** 10.1371/journal.pone.0068885

**Published:** 2013-07-29

**Authors:** Aner Weiss, Anat Mirelman, Aron S. Buchman, David A. Bennett, Jeffrey M. Hausdorff

**Affiliations:** 1 Laboratory for Gait & Neurodynamics, Movement Disorders Unit, Department of Neurology, Tel-Aviv Sourasky Medical Center, Tel-Aviv, Israel; 2 Rush Alzheimer’s Disease Center, Rush University Medical Center, Chicago, Illinois, United States of America; 3 Department of Physical Therapy, Sackler Faculty of Medicine, Tel-Aviv University, Tel-Aviv, Israel; 4 Harvard Medical School, Boston, Massachusetts, United States of America; UCSD School of Medicine, United States of America

## Abstract

**Objective:**

The identification and documentation of subclinical gait impairments in older adults may facilitate the appropriate use of interventions for preventing or delaying mobility disability. We tested whether measures derived from a single body-fixed sensor worn during traditional Timed Up and Go (TUG) testing could identify subclinical gait impairments in community dwelling older adults without mobility disability.

**Methods:**

We used data from 432 older adults without dementia (mean age 83.30±7.04 yrs, 76.62% female) participating in the Rush Memory and Aging Project. The traditional TUG was conducted while subjects wore a body-fixed sensor. We derived measures of overall TUG performance and different subtasks including transitions (sit-to-stand, stand-to-sit), walking, and turning. Multivariate analysis was used to compare persons with and without mobility disability and to compare individuals with and without Instrumental Activities of Daily Living disability (IADL-disability), all of whom did not have mobility disability.

**Results:**

As expected, individuals with mobility disability performed worse on all TUG subtasks (p<0.03), compared to those who had no mobility disability. Individuals without mobility disability but with IADL disability had difficulties with turns, had lower yaw amplitude (p<0.004) during turns, were slower (p<0.001), and had less consistent gait (p<0.02).

**Conclusions:**

A single body-worn sensor can be employed in the community-setting to complement conventional gait testing. It provides a wide range of quantitative gait measures that appear to help to identify subclinical gait impairments in older adults.

## Introduction

Gait impairment and mobility disability are common in older adults. These alterations in motor function are associated with adverse health consequences, hospitalization, institutionalization and loss of independence. These problems and are therefore a growing public health concern. Tests that can identify older adults in the community-setting who have subclinical mobility impairment offer the opportunity for interventions that can prevent or delay the development of mobility disability, potentially promoting independence among our aging population.

Conventional gait testing of older adults in the community setting and in population-based studies often assesses how long it takes the participant to complete a standardized performance (e.g., timed walk or Timed Up and Go, TUG [1]). These tests have been shown to predict a wide range of adverse health outcomes [2]–[7]. However, they do not provide any information on or determine the specific aspects of gait and mobility that might be impaired. Thus they are limited in their ability to track subtle changes or to target therapy and early interventions. Rapid advances in technology have led to the development of unobtrusive portable equipment that has the capacity to measure both acceleration and angular velocity in 3 directions. Employing these new devices during conventional gait testing could potentially provide a wide range of objective measures of multiple aspects of gait that are not currently captured in the community-setting [1]. Initial pilot work has demonstrated the ability of using instrumented tests such as the TUG in different populations [8]–[13], however, large scale studies in the community setting are still lacking.

We aimed to test the hypothesis that quantitative gait measures obtained during conventional TUG testing could be used to identify subclinical gait impairments in community-dwelling older adults, and more specifically to determine which aspects of mobility are impaired.

To assess the potential added value of using the instrumented TUG, rather than just time to completion of the TUG, we analyzed data from 432 older adults without dementia who were participating in the Rush Memory and Aging Project, a community-based, cohort study of chronic conditions of aging [14]. We utilized a single small, light-weight sensor worn on a belt that measured both acceleration and angular velocity in 3 directions. These data were used to quantify 5 subtasks which comprise the standard TUG performance. These subtasks include: walking, 2 transitions: sit-to-stand and stand-to-sit, and 2 turns: at the middle and at the end of the TUG, before sitting back down. The total TUG time provides a good overall measure. However, it does not shed light on the specific aspects of mobility that are impaired. A priori, the subtasks of the TUG may behave differently. For example, there is evidence suggesting that the demands of straight walking and curved walking (i.e., turns) differ [15], [16]. Similarly, while walking and turning both share features related to locomotion (e.g., reciprocal and rhythmic activation of the left and right lower legs), sit-to-stand abilities are largely related to strength and power of the lower extremities [17], [18]. Indeed, previous work has shown that instrumented assessment of these different subtasks, e.g., transitions [19]–[23] or turns [24], may be sensitive to specific gait and balance impairments that cannot be identified using only the TUG duration. Two hypothetical subjects may take the same amount of time to complete the TUG, whereas one requires extra time for sit-to-stand and the other for turning. These differences and the quality of the performance of the individual subtasks cannot be ascertained using only the time to complete the TUG.

In this study, a more comprehensive analysis approach was used to obtain information on all TUG subtasks (as opposed to just the traditional time to completion). In the first part of this study, we compared gait measures in participants with and without self-reported mobility disability to demonstrate the validity of these measures. Then, to examine if these measures could be used to identify subclinical gait impairments, we investigated whether these quantitative gait measures differed in participants with and without Instrumental Activities of Daily Living disability (IADL disability) among the subjects who did not report mobility disability. The IADL scale reflects an individual’s ability to function independently in the community setting. We hypothesized that the instrumented TUG measures would show differences between these two subgroups, perhaps related to specific components of the TUG, even though on the surface, all subjects have relatively intact motor and cognitive function. If this hypothesis is substantiated, then it would suggest that these measures are sensitive to underlying subtle mobility changes, not otherwise observed, and that the instrumented TUG can provide insight into the specific features that are altered. Such a finding may set the stage for detecting mobility disability in its early stages while it is still largely amenable to therapy.

## Methods

### Ethics

All participants signed an informed consent agreeing to annual clinical evaluation. The study was in accordance with the latest version of the Declaration of Helsinki and was approved by the institutional review board of Rush University Medical Center.

### Subjects

All participants were from the Rush Memory and Aging Project (MAP), a longitudinal cohort studies of chronic conditions of old age which began in 1997 [14], [25]. Participants were recruited from retirement facilities and subsidized housing facilities from around the Chicago metropolitan area. The Hybrid body worn sensor (see below) was added in 2011. Persons were eligible for these analyses if they were ambulatory and without clinical dementia at the time of gait testing with the body-fixed sensor.

### Clinical Testing and Clinical Diagnoses

Subjects underwent a uniform structured clinical evaluation including a medical history, neurological examination, motor and cognitive performance testing. Details of the clinical evaluation have been described elsewhere [14], [25]. Trained technicians administered 21 cognitive tests, 19 of which were converted to Z scores and averaged to yield a composite measure of global cognition. Dementia was diagnosed in a three-step process. Cognitive testing was scored by a computer and reviewed by a neuropsychologist to diagnose cognitive impairment. Participants were then evaluated by a physician who used all cognitive and clinical data to diagnose dementia and other common neurological conditions.

### Assessment of Gait: Using the Timed Up and Go (TUG) Test

While wearing a body-fixed sensor, participants underwent conventional TUG testing. Participants were instructed as follows: “You’re going to stand up from this chair, walk to the other end of the course at your normal pace past the line, turn around, walk back to the chair and sit down”. During testing, participants wore a portable small, light-weight body-fixed sensor (Hybrid, Mcroberts) on a neoprene belt placed on their lower back at the level of anterior iliac crest. The sensor weighs 74 gm and its dimensions are (87×45×14 mm).

The Hybrid includes a triaxial accelerometer (sensor range and resolution are: ±2 g and ±1 mg respectively) and a triaxial gyroscope (sensor range and resolution are: ±100 deg/s and ±0.0069 deg/s, respectively). Altogether 6 acceleration and angular velocity signals are recorded continuously during TUG testing. Signals include 3 acceleration axes: Vertical acceleration (V), medio-lateral acceleration (ML), anterior-posterior acceleration (AP); and 3 angular velocity axes: yaw- which is the rotation around the V axis, Pitch- which is the rotation around the ML axis, and Roll- which is the rotation around the AP axis.

The device was set to record continuously during 2 trials of TUG testing. The data which was recorded was saved on a Secure Digital (SD) card at a sample frequency of 100 Hz. After testing was completed the data was transferred to a personal computer for further analysis (using Matlab, the Mathworks software).

### Quantifying TUG Subtasks

Quantitative gait measures were derived from the second TUG trial. An automated algorithm for detecting the start and end times of the TUG based on the AP axis was used to derive the overall time that it took for the participant to complete the TUG, as previously described [11]. We derived quantitative gait measures for 5 subtasks from the 2^nd^ TUG trial. The measures from these five subtasks are described below.

#### Transition subtasks

**Figure 1:**
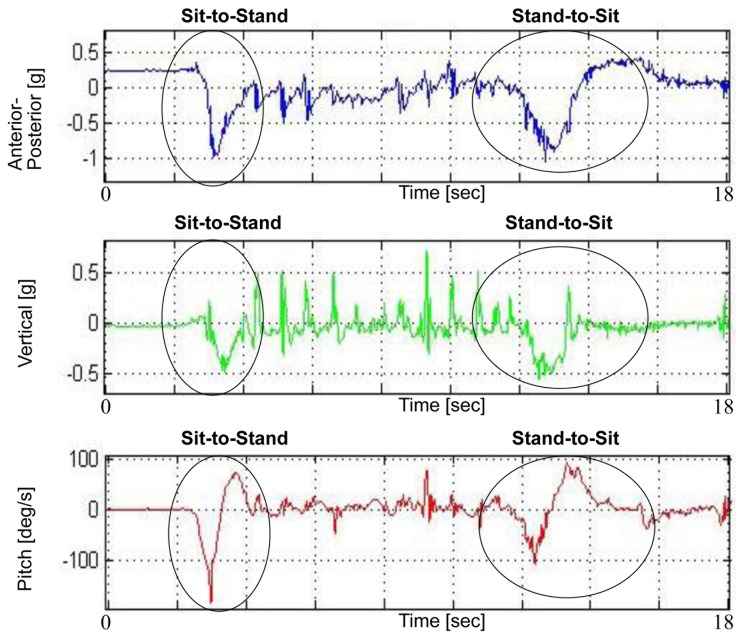
Transition components. Transition components derived from the anterior-posterior, vertical, and pitch axes.

Measures from two transitions: sit to stand (transition 1) and the stand to sit (transition 2), were derived from the AP, V and pitch axes (Figure 1). The extraction of the AP measures is described elsewhere [10], [11], and includes the duration of each transition as well as range and jerk measures, median and standard deviation values. Here, we extracted the same measures from the V axis in order to provide information related to leg muscle strength and balance during the transitions [26].

**Figure 2:**
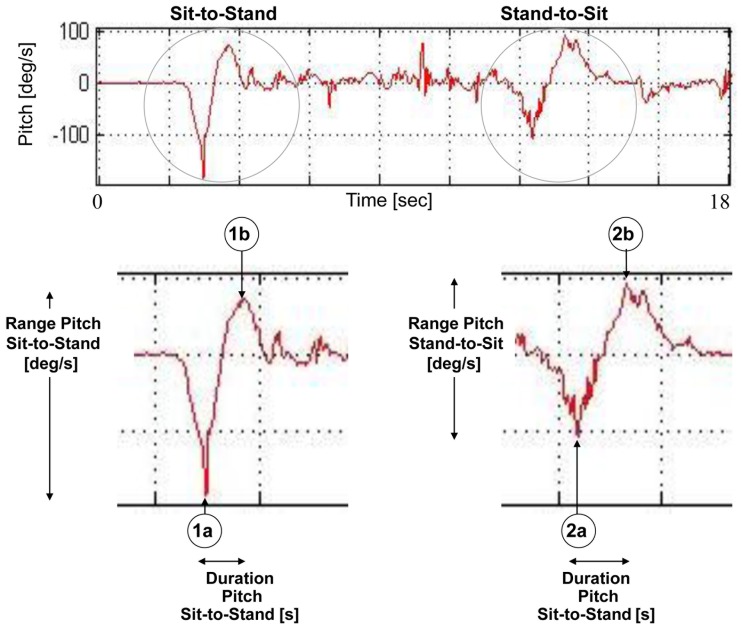
Transition measures from the pitch axis. Transition components derived from the pitch axis. The upper figure shows the TUG pitch signal, where transition 1 (sit-to-stand) and transition 2 (stand-to-sit) components are evident as “tilted S” shaped objects. The lower figure shows a close-up of pitch transitions, with 1a and 1b as the minimum and maximum points of transition 1 (respectively), and 2a and 2b as the minimum and maximum points of transition 2, respectively. The pitch range [deg/s] and the transition duration [s] measures are depicted. The jerk, which is not shown here, is simply the slope of the pitch signal between the minimum and maximum points.

The pitch measures which are shown in figure 2 include the range, jerk, and duration of each transition. As seen in figure 2, both transitions look like “tilted S” shaped objects. The “lean to rise” part of transition 1 is shown as a “concave”, and the “active rise” part of transition 1 is shown as an “upside down concave”. Similarly, the “lean to sit” part of transition 2 is shown as a “concave”, and the “active sit” part of transition 2 looks like an “upside down concave”.

Points 1a and 1b (figure 2) were determined as the minimum and maximum points of the pitch signal of transition 1, and points 2a and 2b (figure 2) were determined as the minimum and maximum points of the pitch signal of transition 2. The duration pitch measure was therefore composed as the duration from point 1a (or 2a) to point 1b (or 2b). Similarly, the range pitch measure was composed as the range of the pitch signal from the maximum 1b (or 2b) point to the minimum 1a (or 2a) point. The Jerk was determined as the estimated slope of the pitch signal between these maximum and minimum points. Additional transition measures included the range of the acceleration in all 6 axes, in both transitions.

#### Walking subtasks

**Figure 3:**
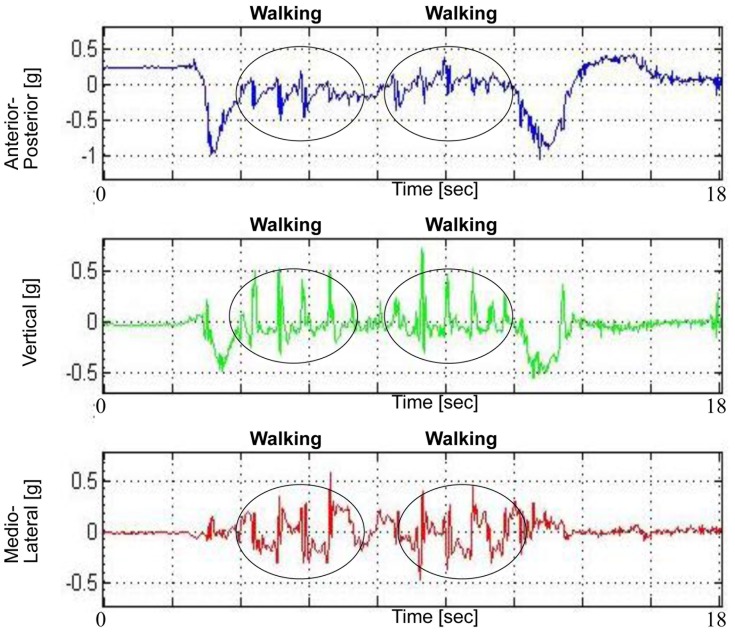
Walking components. Walking components derived from the anterior-posterior, vertical, and medio-lateral axes.

Walking measures were derived from the V, AP and ML axes and excluded the segments which included the 2 transitions and 2 turning subtasks (Figure 3). The initial walking (walk 1) subtask included the interval from the end of the sit-to-stand (determined from the AP axis) until the beginning of the 1st turn (determined from the yaw axis). Walk 1 was concatenated with the interval from the end of the 1^st^ turn (determined from the yaw axis) until the beginning of the turn-to-sit (determined from the AP/yaw axis) (referred to as walk 2). The rationale for concatenating walk 1 and 2 (referred to as walking portions), rather than assessing their measures separately, was because each segment alone was too short for determining valid gait measures. Also, we assumed that there is not much difference in the gait measures of both walking segments. Walking measures included the total walking duration as well as number of steps taken. Step and stride regularity were derived from the autocorrelation signal of the V, AP and ML walking portion [27]. Step duration was also determined from the V signal. Additional measures derived from the gait portion included the range of the accelerations and angular velocities in all 6 axes.

#### Turning subtasks

**Figure 4:**
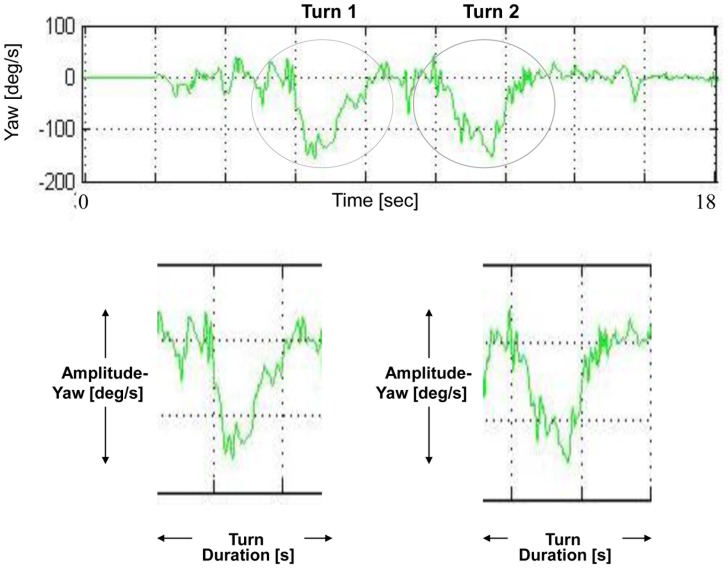
Turn component. Turn component derived from the yaw axis. The upper figure shows the TUG yaw signal, where turn 1 and turn 2 components are evident as “concave” shaped objects. The lower figure shows a close-up of both yaw turns, depicting the turn yaw amplitude [deg/s] and the turn duration [s] measures (number of steps during the turns is not showed here, as it is derived from the vertical axis).

Turning measures were derived from the yaw axis. As seen in figure 4, the 1^st^ and 2^nd^ turn components are evident in the yaw signal as 2 high amplitude peaks in the signal. The 1^st^ peak represents the turn performed in the middle of the TUG, and the 2^nd^ peak represents the turn performed at the end of the trial, before sitting back down.

The start and end points of each turn were determined as the points in the yaw signal in which it crossed 0.1 of the maximum yaw peak amplitude of the turn. The yaw turn amplitude measure was determined as the amplitude of the yaw peak in its absolute value. The direction of the “concave” shape (facing up or down) determines the direction of the turn (left or right). Since in the scope of this work we were not interested in the turning direction, the yaw amplitude measures were taken as their absolute value.

Turn duration was determined as the duration from the beginning until the end of the turn, and number of steps during the turn was derived from the vertical axis and defined as the number of steps in the vertical axis performed from the beginning until the end of the turn. Additional turn measures included the range of the acceleration in all 6 axes, in both turns.

We checked the reliability of the derived measures by performing Pearson’s correlations between the acceleration and angular velocity measures extracted from the 1^st^ TUG trial and the same measures extracted from the 2^nd^ TUG trial. Despite possible learning or practice effects, all measures were significantly correlated between the two trials (p<0.0001). For example, the correlation coefficient was larger than 0.80 for the turn yaw amplitude and step duration.

### Self-Report Assessment of Mobility Disability

#### The rosow-breslau scale

[28], is an indicator of a person’s functional health status, and was used to assess mobility disability. It focuses on three tasks that require mobility and strength: walking up and down a flight of stairs, walking half a mile, and doing heavy housework like washing windows, walls, or floors. The participant was asked if he/she could perform each task without help. Any person needing help with one or more tasks were classified as disabled.

#### Instrumental Activities of Daily Living (IADLs)

Refers to daily activities performed in an individual’s home, natural setting that are essential for living independently in the community. The IADL scale is therefore used as an indicator of a person’s ability or inability to function independently in his or her natural environment. IADLs were assessed using 8 items adapted from the Duke Older Americans Resources and Services project [29]. Participants were asked to rate their ability to perform (no help, help, unable to do) eight instrumental activities of daily living that are cognitively demanding: telephone use, meal preparation, money management, medication management, light and heavy housekeeping, shopping, and local travel. Participants needing help with (i.e., dependency) or unable to perform one or more tasks, were classified as having disability.

### Other Covariates

Demographic information including date of birth, gender, and years of education, were collected via participant interview.

### Statistical Analysis

Binary logistic regression tests adjusted for age and sex were performed in order to compare gait measures from the participants with and without mobility disability. We also assessed the gait measures of participants without mobility disability, and compared between the participants with and without IADL disability. The p-values were corrected for multiple comparisons using the widely used method of Benjamini-Hochberg [30]. Group values are reported as mean±standard deviation. Statistical analyses were carried out using SPSS version 19.

## Results

There were 432 persons in these analyses. 203 subjects had mobility disability and 229 did not have mobility disability (see Table S1 in Appendix S1 for subject characteristics). From the 229 with no mobility disability, 52 had IADL disability and 177 were with no IADL disability.

### Quantitative Gait Measures in Older Adults with and without Mobility Disability

As shown in Appendix S1 and as expected, many measures from all TUG components differed between persons with and without mobility disability. People with mobility disability took longer to complete the TUG (14.88±4.29 sec vs. 11.12±2.49 sec; p<0.0001), exhibited longer walking duration (8.44±2.89 sec vs. 5.95±1.55 sec; p<0.0001), longer step duration, and lower gait consistency (Table S2a in Appendix S1). People with mobility disability also took longer to complete the transitions and turns, and exhibited lower acceleration amplitudes during the turns (Table S2b, S2c, S2d in Appendix S1).

### Quantitative Gait Measures in Participants with and without IADL Disability but without Mobility Disability

For these analyses, we included only participants without mobility disability and examined whether there were differences in gait performance between participants with and without IADL disability (77.3% without IADL disability). Subject characteristics are shown in table 1.

**Table 1:** Characteristics of subjects with and without IADL disability.

Measures	IADLdisability	No IADLdisability	P-value
# of subjects (N)	52	177	–
Age (yrs)	84.01±6.92	81.12±7.23	0.011
Gender (% women)	80.76%	66.10%	0.047
Height (m)	1.61±0.08	1.66±0.16	0.0081
Weight (kg)	68.29±13.86	73.82±14.35	0.016
Body-mass-index (kg/mˆ2)	25.99±4.45	26.67±4.95	0.381
Years of education	15.15±3.20	15.51±3.04	0.452
Global cognitive Score	−0.09±0.75	0.34±0.51	<0.001

#### Subject characteristics

Subjects with IADL disability were about 3 years older than subjects without IADL disability, were more likely to be female, were shorter and weighed less, and had nearly a half standard unit lower cognitive function scores (table 1). The groups did not differ with respect to years of education.

#### Overall TUG component

Those with IADL disability took about 25% more time to complete the TUG (13.59±4.21 sec vs. 10.88±2.51 sec; p<0.0001) (table 2).

**Table 2:** TUG signal derived measures of walking in subjects with and without IADL disability.

Measures		IADL disability	No IADL disability	P-value
**Overall TUG** (including all components)				
TUG duration [s]		13.59±4.21	10.88±2.51	<0.0001*
**Walking component** (after removing turns)				
Duration of entire walking portion [s]		7.51±2.97	5.88±1.63	<0.0001*
Number of steps	V	12.70±4.40	9.90±2.35	<0.0001*
Step regularity [g^∧^2]	V	0.49±0.13	0.53±0.14	0.054
	AP	0.46±0.14	0.48±0.14	0.253
	ML	−0.37±0.16	−0.34±0.12	0.262
Stride regularity [g^∧^2]	V	0.39±0.14	0.44±0.15	0.026
	AP	0.37±0.13	0.44±0.15	0.015*
	ML	0.34±0.14	0.31±0.11	0.177
Step duration [s]	V	59.57±5.02	58.98±6.74	0.402
Range	V	0.96±0.23	1.12±0.26	0.004*
	AP	0.86±0.161	0.92±0.20	0.516
	ML	0.65±0.19	0.78±0.26	0.088
	YAW	92.88±25.33	96.76±24.20	0.187
	PITCH	104.93±29.34	115.16±36.88	0.727
	ROLL	62.24±20.21	70.62±24.84	0.062

* indicates those measures that significantly differed in the two groups, after correcting for multiple comparisons using the Benjamini-Hochberg method (i.e., using a threshold of p = 0.015). All entries are adjusted for age and gender.

#### Walking component

Subjects with IADL disability exhibited significantly longer walking duration (7.51±2.97 sec vs. 5.88±1.63 sec; p<0.0001), higher number of steps to complete the TUG (12.70±4.40 sec vs. 9.90±2.35 sec; p<0.0001), and lower AP stride-regularity, compared to the non-impaired IADL group (table 2).

#### Transitioning- sit-to-stand and stand-to-sit

Interestingly, no differences were observed in the transition measures of both groups.

#### Turning 1 and 2 components

Subjects with IADL disability exhibited higher turn duration and lower yaw angular velocity amplitude and acceleration ranges during both of the turns (table 3).

**Table 3:** TUG signal derived measures of turning during the two turns in subjects with and without IADL disability.

Measures		IADLdisability	No IADLdisability	P-value*
**Turn 1** (middle of TUG)				
Amplitude-yaw [deg/s]		145.78±36.05	165.56±35.48	0.004*
Turn duration [s]		2.31±0.57	1.99±0.46	0.001*
Number of steps	V	4.67±1.42	3.90±1.48	0.010*
Range [g]	V	0.55±0.23	0.70±0.28	0.011*
	AP	0.37±0.13	0.43±0.11	0.008*
	ML	0.55±0.17	0.68±0.22	0.003*
	YAW	133.71±34.00	152.97±32.48	0.003*
	PITCH	62.55±27.18	67.17±23.40	0.239
	ROLL	51.31±16.85	66.02±26.50	0.034*
**Turn 2** (end of TUG)				
Amplitude-yaw [deg/s]		142.67±43.38	166.69±34.82	0.001*
Turn duration [s]		2.33±0.70	1.91±0.46	<0.0001*
Number of steps	V	3.99±2.16	3.18±1.26	0.003*
Range	V	0.67±0.22	0.81±0.29	0.012*
	AP	0.60±0.18	0.64±0.16	0.274
	ML	0.60±0.15	0.71±0.22	0.087
	YAW	132.27±39.39	154.30±31.33	0.001*
	PITCH	80.09±28.01	84.05±23.93	0.820
	ROLL	61.98±23.89	71.04±23.85	0.060

* indicates those measures that significantly differed in the two groups, after correcting for multiple comparisons using the Benjamini-Hochberg method (i.e., using a threshold of p = 0.034). All entries are adjusted for age and gender.

## Discussion

In a group of more than 400 community-dwelling older persons, a single body-fixed sensor that was worn while subjects completed conventional testing of the TUG, provided a wide range of quantitative gait measures. Some of these gait measures were related with self-reported mobility disability which provides concurrent validity. Others, however were not related to self-reported mobility disability, suggesting that these measures capture additional aspects of gait and mobility [31]. Further analyses of individuals without self-reported mobility disability supports the intriguing possibility that individuals with IADL disability may have subclinical gait impairment, especially for turning. Employing a body-fixed sensor during conventional gait testing does not increase participant testing burden and provides a more comprehensive description of gait performance in older adults. Metrics derived from the instrumented TUG apparently can help to identify the specific components of the task that may be impaired and are in need of therapy, adding clinically relevant information beyond time to completion.

Although catastrophic medical events can cause the rapid onset of mobility disability, more commonly, it develops gradually over time with progressive gait impairments in the absence of overt clinical disease. Even in the absence of clinical complaints of walking difficulties, behavioral adaptations, i.e., changing the way one usually carries out a task, may be an early indicator of gait impairment. These adaptations are associated with incident mobility disability. Thus, tests which can identify subclinical gait impairments and individuals at risk for mobility disability are essential for public health efforts to decrease the burden of mobility disability in our aging population. Walking occurs in three dimensional space and requires the production of coordinated rhythmic patterns of multiple muscles, the postural control of the moving body, and the adaptation of these movements to motivational and environmental demands. Accumulating evidence suggests that distinct neural systems control different aspects of gait and mobility that can cause a wide variety of gait impairments [32]–[35]. However, our increased understanding of the brain mechanisms that underlie mobility has not yet been fully translated into the clinical domain or into large scale epidemiologic cohort studies. In part, this is due to the lack of portable equipment that can be used to quantify the various subtasks which underlie the different gait performances tested in the community-setting. The current study employed unobtrusive portable instrumentation during TUG testing to provide a wide variety of quantitative measures of the TUG subtasks not currently captured with conventional gait testing of older adults in the community setting. Indeed, while time to complete the TUG was significantly longer in subjects with IADL disability, compared to those that did not have IADL disability, the instrumented TUG allows for teasing out and identifying the specific features of mobility that were altered.

Prior studies have demonstrated the utility of adding instrumentation to traditional gait testing, but these have required testing in the laboratory setting [36], [37]. This has led to gaps in our knowledge with respect to the characterization of the full spectrum of gait impairments in older adults, especially among older more debilitated individuals, who are unable to participate in laboratory studies [38]. The present study leveraged advances in technology making it possible to collect quantitative measures of gait in the community setting. These devices are minimally intrusive or burdensome to participants and have the distinct advantage of simultaneously measuring 3 dimensional changes in both acceleration and angular velocity [39], [40]. Measuring both simultaneously offers the possibility to quantify not only traditional spatiotemporal measures of gait, but also angles and angular velocity of various body segments during gait testing.

The current study found that people with mobility disability are impaired in all 5 TUG subtasks. Their transitions, walking and turning are slower, their gait variability is higher, transition jerks are lower, and acceleration ranges in all subtasks are lower. Together the measures collected in the current study provide a more comprehensive description of the clinical gait phenotype in older adults with and without mobility disability. Further work will be needed to determine which mobility related brain networks control the TUG subtasks and whether these subtasks decline at different rates, are differentially affected by risk factors and pathologies, or associated with different adverse outcomes [41].

The present findings not only demonstrates the feasibility of enhancing conventional TUG testing in community-based studies of older individuals, but also extends prior studies [42], [43] by showing subclinical gait impairments particularly for turning in individuals with IADL disability. Interestingly, sit-to-stand and stand-to-sit were not different in the subjects with or without IADL disability. Only specific aspects of the TUG differed between these two groups, i.e., walking and turning. The link between IADL disability and turning may be due to the fact that turning may be more cognitively demanding and requires a larger degree of planning, orientation in space and organization [16], [44]. Nonetheless, the present results suggest the potential utility of employing a single body-fixed sensor during conventional gait testing in *asymptomatic* older adults to identify subclinical gait impairments. This may allow for the possibility of earlier intervention to prevent or delay the development of mobility disability in older adults and thus decrease the burden of this growing public health challenge.

### Limitations and Future Work

Future work can be done in dimensionality reduction in order to obtain less redundancy of measures, and a more simplified, straight-forward clinical interpretation of the various TUG subtasks and measures. The current work is cross sectional and it will be important to investigate the relationship of the TUG subtasks measures with respect to the subsequent development of falls, their relationship to cognitive impairments and the development of mobility disability.

## Supporting Information

Appendix S1 **Validation: Quantitative gait measures in older adults with and without mobility disability. Table S1. Table S2.**
(DOCX)

## References

[1] Podsiadlo D, Richardson S (1991) The timed “Up & Go”: a test of basic functional mobility for frail elderly persons. J Am Geriatr Soc 39: 142–148. 1991946 10.1111/j.1532-5415.1991.tb01616.x

[2] Tangen GG, Londos E, Olsson J, Minthon L, Mengshoel AM (2012) A longitudinal study of physical function in patients with early-onset dementia. Dement Geriatr Cogn Dis Extra 2: 622–631 10.1159/000345782 [doi];dee-0002-0622 [pii]. 23341827 10.1159/000345782 PMC3551435

[3] Donoghue OA, Horgan NF, Savva GM, Cronin H, O’Regan C, et al (2012) Association between timed up-and-go and memory, executive function, and processing speed. J Am Geriatr Soc 60: 1681–1686 10.1111/j.1532-5415.2012.04120.x [doi]. 22985141 10.1111/j.1532-5415.2012.04120.x

[4] Bossers WJ, van der Woude LH, Boersma F, Scherder EJ, van Heuvelen MJ (2012) Recommended measures for the assessment of cognitive and physical performance in older patients with dementia: a systematic review. Dement Geriatr Cogn Dis Extra 2: 589–609 10.1159/000345038 [doi];dee-0002-0589 [pii]. 23341825 10.1159/000345038 PMC3551396

[5] Idland G, Engedal K, Bergland A (2013) Physical performance and 13.5-year mortality in elderly women. Scand J Public Health 41: 102–108. 23178925 10.1177/1403494812466460

[6] Viccaro LJ, Perera S, Studenski SA (2011) Is timed up and go better than gait speed in predicting health, function, and falls in older adults? J Am Geriatr Soc 59: 887–892. 21410448 10.1111/j.1532-5415.2011.03336.x PMC3522463

[7] Wennie Huang WN, Perera S, VanSwearingen J, Studenski S (2010) Performance measures predict onset of activity of daily living difficulty in community-dwelling older adults. J Am Geriatr Soc 58: 844–852. 20406319 10.1111/j.1532-5415.2010.02820.x PMC2909370

[8] Salarian A, Horak FB, Zampieri C, Carlson-Kuhta P, Nutt JG, et al (2010) iTUG, a sensitive and reliable measure of mobility. IEEE Trans Neural Syst Rehabil Eng 18: 303–310. 20388604 10.1109/TNSRE.2010.2047606 PMC2922011

[9] Spain RI, St George RJ, Salarian A, Mancini M, Wagner JM, et al (2012) Body-worn motion sensors detect balance and gait deficits in people with multiple sclerosis who have normal walking speed. Gait Posture 35: 573–578. 22277368 10.1016/j.gaitpost.2011.11.026 PMC3614340

[10] Weiss A, Herman T, Plotnik M, Brozgol M, Maidan I, et al (2010) Can an accelerometer enhance the utility of the Timed Up & Go Test when evaluating patients with Parkinson’s disease? Med Eng Phys 32: 119–125. 19942472 10.1016/j.medengphy.2009.10.015

[11] Weiss A, Herman T, Plotnik M, Brozgol M, Giladi N, et al (2011) An instrumented timed up and go: the added value of an accelerometer for identifying fall risk in idiopathic fallers. Physiol Meas 32: 2003–2018. 22094550 10.1088/0967-3334/32/12/009

[12] Zampieri C, Salarian A, Carlson-Kuhta P, Aminian K, Nutt JG, et al (2010) The instrumented timed up and go test: potential outcome measure for disease modifying therapies in Parkinson’s disease. J Neurol Neurosurg Psychiatry 81: 171–176. 19726406 10.1136/jnnp.2009.173740 PMC3065923

[13] Zampieri C, Salarian A, Carlson-Kuhta P, Nutt JG, Horak FB (2011) Assessing mobility at home in people with early Parkinson’s disease using an instrumented Timed Up and Go test. Parkinsonism Relat Disord 17: 277–280. 20801706 10.1016/j.parkreldis.2010.08.001 PMC2995832

[14] Bennett DA, Schneider JA, Buchman AS, Mendes de LC, Bienias JL, et al (2005) The Rush Memory and Aging Project: study design and baseline characteristics of the study cohort. Neuroepidemiology 25: 163–175. 16103727 10.1159/000087446

[15] Odonkor CA, Thomas JC, Holt N, Latham N, Vanswearingen JM, et al.. (2013) A Comparison of Straight- and Curved-Path Walking Tests Among Mobility-Limited Older Adults. J Gerontol A Biol Sci Med Sci. 10.1093/gerona/glt060 PMC3814235 23657972

[16] Lowry KA, Brach JS, Nebes RD, Studenski SA, VanSwearingen JM (2012) Contributions of cognitive function to straight- and curved-path walking in older adults. Arch Phys Med Rehabil 93: 802–807. 22541307 10.1016/j.apmr.2011.12.007 PMC4878139

[17] Bernardi M, Rosponi A, Castellano V, Rodio A, Traballesi M, et al (2004) Determinants of sit-to-stand capability in the motor impaired elderly. J Electromyogr Kinesiol 14: 401–410 10.1016/j.jelekin.2003.09.001 [doi];S1050641103001081 [pii]. 15094153 10.1016/j.jelekin.2003.09.001

[18] Takai Y, Ohta M, Akagi R, Kanehisa H, Kawakami Y, et al.. (2009) Sit-to-stand test to evaluate knee extensor muscle size and strength in the elderly: a novel approach. J Physiol Anthropol 28: 123–128. JST.JSTAGE/jpa2/28.123 [pii]. 10.2114/jpa2.28.123 19483373

[19] Doheny EP, Fan CW, Foran T, Greene BR, Cunningham C, et al (2011) An instrumented sit-to-stand test used to examine differences between older fallers and non-fallers. Conf Proc IEEE Eng Med Biol Soc 2011: 3063–3066. 22254986 10.1109/IEMBS.2011.6090837

[20] Janssen WG, Kulcu DG, Horemans HL, Stam HJ, Bussmann JB (2008) Sensitivity of accelerometry to assess balance control during sit-to-stand movement. IEEE Trans Neural Syst Rehabil Eng 16: 479–484. 18990651 10.1109/TNSRE.2008.2003386

[21] Najafi B, Aminian K, Loew F, Blanc Y, Robert PA (2002) Measurement of stand-sit and sit-stand transitions using a miniature gyroscope and its application in fall risk evaluation in the elderly. IEEE Trans Biomed Eng 49: 843–851. 12148823 10.1109/TBME.2002.800763

[22] Salarian A, Russmann H, Vingerhoets FJ, Burkhard PR, Aminian K (2007) Ambulatory monitoring of physical activities in patients with Parkinson’s disease. IEEE Trans Biomed Eng 54: 2296–2299. 18075046 10.1109/tbme.2007.896591

[23] Zijlstra A, Mancini M, Lindemann U, Chiari L, Zijlstra W (2012) Sit-stand and stand-sit transitions in older adults and patients with Parkinson’s disease: event detection based on motion sensors versus force plates. J Neuroeng Rehabil 9: 75. 23039219 10.1186/1743-0003-9-75 PMC3546014

[24] Salarian A, Zampieri C, Horak FB, Carlson-Kuhta P, Nutt JG, et al (2009) Analyzing 180 degrees turns using an inertial system reveals early signs of progression of Parkinson’s disease. Conf Proc IEEE Eng Med Biol Soc 2009: 224–227. 19964471 10.1109/IEMBS.2009.5333970 PMC2954632

[25] Bennett DA, Schneider JA, Buchman AS, Barnes LL, Boyle PA, et al.. (2012) Overview and findings from the rush Memory and Aging Project. Curr Alzheimer Res 9: 646–663. CAR-EPUB-20120402-008 [pii]. 10.2174/156720512801322663 PMC3439198 22471867

[26] Zijlstra W, Bisseling RW, Schlumbohm S, Baldus H (2010) A body-fixed-sensor-based analysis of power during sit-to-stand movements. Gait Posture 31: 272–278. 19963386 10.1016/j.gaitpost.2009.11.003

[27] Moe-Nilssen R, Helbostad JL (2004) Estimation of gait cycle characteristics by trunk accelerometry. J Biomech 37: 121–126. 14672575 10.1016/s0021-9290(03)00233-1

[28] Rosow I, Breslau N (1966) A Guttman health scale for the aged. J Gerontol 21: 556–559. 5918309 10.1093/geronj/21.4.556

[29] Lawton MP, Brody EM (1969) Assessment of older people: self-maintaining and instrumental activities of daily living. Gerontologist 9: 179–186. 5349366

[30] Benjamini Y, Hochberg Y (1995) Controlling the false discovery rate: a practical and powerful approach to multiple testing. Journal of the Royal Statistical Society 57 (1): 125–133.

[31] Bean JF, Olveczky DD, Kiely DK, LaRose SI, Jette AM (2011) Performance-based versus patient-reported physical function: what are the underlying predictors? Phys Ther 91: 1804–1811. 22003163 10.2522/ptj.20100417 PMC3229045

[32] Grillner S (2011) Neuroscience. Human locomotor circuits conform. Science 334: 912–913. 22096178 10.1126/science.1214778

[33] Ivanenko YP, Poppele RE, Lacquaniti F (2006) Motor control programs and walking. Neuroscientist 12: 339–348. 16840710 10.1177/1073858406287987

[34] Petersen TH, Willerslev-Olsen M, Conway BA, Nielsen JB (2012) The motor cortex drives the muscles during walking in human subjects. J Physiol 590: 2443–2452. 22393252 10.1113/jphysiol.2012.227397 PMC3424763

[35] van den BR, Heutschi J, Barraud Q, DiGiovanna J, Bartholdi K, et al (2012) Restoring voluntary control of locomotion after paralyzing spinal cord injury. Science 336: 1182–1185. 22654062 10.1126/science.1217416

[36] Brach JS, Studenski SA, Perera S, VanSwearingen JM, Newman AB (2007) Gait variability and the risk of incident mobility disability in community-dwelling older adults. J Gerontol A Biol Sci Med Sci 62: 983–988. 17895436 10.1093/gerona/62.9.983 PMC2858390

[37] Verghese J, Xue X (2011) Predisability and gait patterns in older adults. Gait Posture 33: 98–101. 21050762 10.1016/j.gaitpost.2010.10.004 PMC3052990

[38] Bean JF, Kiely DK, LaRose S, Leveille SG (2008) Which impairments are most associated with high mobility performance in older adults? Implications for a rehabilitation prescription. Arch Phys Med Rehabil 89: 2278–2284. 19061739 10.1016/j.apmr.2008.04.029

[39] Ishigaki N, Kimura T, Usui Y, Aoki K, Narita N, et al (2011) Analysis of pelvic movement in the elderly during walking using a posture monitoring system equipped with a triaxial accelerometer and a gyroscope. J Biomech 44: 1788–1792. 21546026 10.1016/j.jbiomech.2011.04.016

[40] Rouhani H, Favre J, Crevoisier X, Aminian K (2012) Measurement of multi-segment foot joint angles during gait using a wearable system. J Biomech Eng 134: 061006. 22757503 10.1115/1.4006674

[41] Segal NA, Boyer ER, Wallace R, Torner JC, Yack HJ (2012) Association Between Chair Stand Strategy and Mobility Limitations in Older Adults With Symptomatic Knee Osteoarthritis. Arch Phys Med Rehabil. 10.1016/j.apmr.2012.09.026 PMC3847816 23063791

[42] Brach JS, VanSwearingen JM (2002) Physical impairment and disability: relationship to performance of activities of daily living in community-dwelling older men. Phys Ther 82: 752–761. 12147005

[43] Landgraff NC, Whitney SL, Rubinstein EN, Yonas H (2010) Cognitive and physical performance in patients with asymptomatic carotid artery disease. J Neurol 257: 982–991. 20099067 10.1007/s00415-009-5449-z

[44] Herman T, Giladi N, Hausdorff JM (2011) Properties of the ‘timed up and go’ test: more than meets the eye. Gerontology 57: 203–210. 20484884 10.1159/000314963 PMC3094679

