# Supplementary material for: Using a Body-Fixed Sensor to Identify Subclinical Gait Difficulties in Older Adults with IADL Disability: Maximizing the Output of the Timed Up and Go
Source: PLoS One. 2013 Jul 29;8(7):e68885. doi: 10.1371/journal.pone.0068885 (PMC3726691; doi:10.1371/journal.pone.0068885)
Supplement: Appendix S1 — Validation: Quantitative gait measures in older adults with and without mobility disability. Table S1. Table S2. (DOCX) [file pone.0068885.s001.docx]

**Appendix A:**

**Validation: Quantitative Gait Measures in Older Adults with and without mobility disability**

For these analyses, we compared participants without and with self-reported mobility disability (53.11% no disability). Subject characteristics are shown in Table 1. Group comparisons were performed using binary logistic regression, while adjusting for age and sex. The results are shown in Table 2.

***Subject Characteristics:*** Subjects with mobility disability were significantly older than subjects without mobility disability (mobility disability: 84.94±6.40 yrs, No mobility disability: 81.83±7.27 yrs; p<0.0001). Subjects with mobility disability also had higher percent of women (mobility disability: 84.80% women, No mobility disability: 69.65%; p<0.001), and lower height (mobility disability: 1.60±0.10 meters, No mobility disability: 1.65±0.10; p<0.0001). The two groups also differed in their years of education (mobility disability: 14.79±2.81 years, No mobility disability: 15.41±3.08 years; p=0.030) and global cognitive score (mobility disability: 0.125±0.48 meters, No mobility disability: 0.23±0.61; p=0.038).

***Overall TUG component:*** As expected, individuals with mobility disability took longer to complete the TUG (p<0.0001) (see Table 2a).

***Walking component:*** The mobility disability group exhibited significantly longer walking duration, higher step duration, higher number of steps, and higher gait variability compared to the group without mobility disability (see Table 2a).

***Sit-to-Stand and Stand-to-Sit Transitions:*** The mobility disability group exhibited longer transition durations in both transitions and lower jerks in the stand-to-sit transition (see Table 2b and 2c).

***Turning 1 and 2 components:*** The mobility disability group exhibited significantly higher turn duration and lower yaw amplitude and acceleration ranges during both turns (see Table 2d).

| **Table 1:**  Characteristics of subjects with and without mobility disability | | | |
| --- | --- | --- | --- |
| **Measures** | **mobility disability** | **No mobility disability** | **P-value** |
| # of subjects (N) | 203 | 229 | -- |
| Age (yrs) | 84.94±6.40 | 81.83±7.27 | <0.0001 |
| Gender (% women) | 84.80% | 69.65% | <0.001 |
| Height (m) | 1.60±0.10 | 1.65±0.10 | <0.0001 |
| Weight (kg) | 70.84±16.56 | 72.59±14.40 | 0.248 |
| Body-mass-index (kg/m2) | 27.55±5.95 | 26.52±4.84 | 0.050 |
| Years of education | 14.79±2.81 | 15.41±3.08 | 0.030 |
| Global cognitive Score | 0.13±0.48 | 0.23±0.61 | 0.038 |

| **Table 2a:** TUG signal derived measures in subjects with and without mobility disability | | | | |
| --- | --- | --- | --- | --- |
| **Measures** | | **mobility disability** | **No mobility disability** | **P-value** |
| **Overall TUG** | | | | |
| TUG duration [s] | | 14.88±4.29 | 11.12±2.49 | <0.0001 * |
| **Walking Component** (after removing turn) | | | | |
| Duration- entire walking portion [s] | | 8.44±2.89 | 5.95±1.55 | <0.0001 * |
| Number of steps | V | 13.48±3.97 | 10.16±2.45 | <0.0001 * |
| Step regularity [g^2] | V | 0.45±0.15 | 0.52±0.13 | <0.0001 * |
|  | AP | 0.41±0.15 | 0.47±0.14 | <0.0001 * |
|  | ML | -0.35±0.17 | -0.35±0.13 | 0.967 |
| Stride regularity [g^2] | V | 0.36±0.13 | 0.43±0.15 | <0.0001 * |
|  | AP | 0.37±0.14 | 0.42±0.15 | 0.003 * |
|  | ML | 0.35±0.14 | 0.31±0.12 | 0.011 * |
| Step duration [s] | V | 61.38±7.16 | 59.12±6.38 | 0.001 * |
| Range | V | 0.90±0.25 | 1.08±0.26 | <0.0001 * |
|  | AP | 0.81±0.16 | 0.89±0.17 | 0.050 |
|  | ML | 0.61±0.17 | 0.75±0.26 | 0.027 |
|  | YAW | 93.05±24.49 | 94.75±22.97 | 0.773 |
|  | PITCH | 92.28±24.28 | 108.79±28.21 | 0.036 * |
|  | ROLL | 60.34±22.39 | 68.65±24.05 | 0.010 * |

*indicates those measures that significantly differed in the two groups, after correcting for multiple comparisons (i.e., using a threshold of p=0.036). All entries are adjusted for age and gender.

| **Table 2b:** TUG signal derived measures during the Sit-to-Stand transition in subjects with and without mobility disability | | | | | |
| --- | --- | --- | --- | --- | --- |
| **Measures** | | | **mobility disability** | **No mobility disability** | **P-value** |
| Duration sit-to-stand [s] | AP | | 0.95±0.48 | 0.67±0.28 | <0.0001 * |
|  | V | | 0.67±0.29 | 0.66±0.32 | 0.001 * |
| Range sit-to-stand [g] | AP | | 0.90±0.23 | 0.920±0.23 | 0.685 |
|  | V | | 0.55±0.26 | 0.55±0.26 | 0.212 |
| Jerk sit-to-stand [g/s] | AP | | -1.09±0.77 | -1.46±0.91 | <0.0001 * |
|  | V | | -0.50±0.75 | -0.26±0.61 | 0.420 |
| Range sit-to-stand A [g] | AP | | 0.49±0.29 | 0.46±0.26 | 0.254 |
|  | V | | 0.43±0.24 | 0.44±0.22 | 0.487 |
| Range sit-to-stand B [g] | AP | | 0.47±0.23 | 0.53±0.26 | 0.492 |
|  | V | | 0.20±0.11 | 0.26±0.13 | 0.593 |
| Jerk sit-to-stand A [g/s] | AP | | -0.97±1.10 | -1.21±1.30 | 0.159 |
|  | V | | -1.11±1.09 | -1.14±0.85 | 0.515 |
| Jerk sit-to-stand B [g/s] | AP | | -1.14±1.01 | -1.61±1.17 | <0.0001 * |
|  | V | | -0.06±0.91 | -0.07±4.46 | 0.613 |
| Median sit-to-stand [g] | AP | | -0.12±0.23 | -0.12±0.22 | 0.821 |
|  | V | | -0.36±0.18 | -0.42±0.17 | 0.002 * |
| SD sit-to-stand [g] | AP | | 0.26±0.07 | 0.27±0.07 | 0.294 |
|  | V | | 0.15±0.09 | 0.15±0.07 | 0.737 |
| Duration pitch sit-to-stand [s] | | | 0.90±0.29 | 0.79±0.25 | <0.0001 * |
| Range pitch sit-to-stand [deg/s] | | | 145.04±40.02 | 170.63±46.46 | 0.013 * |
| Jerk pitch sit-to-stand [deg/s^2] | | | 160.63±90.60 | 217.24±102.39 | <0.0001 * |
| Range sit-to-stand [g] | | ML | 0.23±0.11 | 0.20±0.09 | 0.068 |
|  | | ROLL | 25.01±13.03 | 25.00±12.51 | 0.222 |
|  | | YAW | 33.76±16.72 | 30.93±13.28 | 0.253 |

*indicates those measures that significantly differed in the two groups, after correcting for multiple comparisons (i.e., using a threshold of p=0.013). All entries are adjusted for age and gender.

| **Table 2c:** TUG signal derived measures during the Stand-to-Sit transition in subjects with and without mobility disability | | | | | |
| --- | --- | --- | --- | --- | --- |
| **Measures** | | | **mobility disability** | **No mobility disability** | **P-value** |
| Duration stand -to-sit [s] | AP | | 0.92±0.42 | 0.89±0.45 | 0.008 |
|  | V | | 0.49±0.29 | 0.51±0.24 | 0.062 |
| Range stand -to-sit [g] | AP | | 1.06±0.36 | 0.96±0.21 | 0.046 |
|  | V | | 0.45±0.21 | 0.45±0.20 | 0.314 |
| Jerk stand -to-sit [g/s] | AP | | 1.02±0.56 | 0.99±0.46 | 0.618 |
|  | V | | 0.71±0.60 | 0.54±1.20 | 0.028 |
| Range stand -to-sSit A [g] | AP | | 0.43±0.29 | 0.47±0.26 | 0.304 |
|  | V | | 0.15±0.09 | 0.17±0.09 | 0.441 |
| Range stand -to-sit B [g] | AP | | 0.70±0.43 | 0.60±0.31 | 0.091 |
|  | V | | 0.35±0.17 | 0.36±0.16 | 0.380 |
| Jerk stand -to-sit A [g/s] | AP | | 0.75±0.54 | 0.87±0.59 | 0.864 |
|  | V | | 0.24±0.60 | 0.01±0.61 | 0.026 |
| Jerk stand -to-sit B [g/s] | AP | | 1.15±0.99 | 1.10±1.01 | 0.535 |
|  | V | | 1.19±2.77 | 0.97±2.56 | 0.495 |
| Median stand -to-sit [g] | AP | | -0.15±0.23 | -0.15±0.22 | 0.750 |
|  | V | | -0.34±0.21 | -0.40±0.17 | 0.003 * |
| SD stand -to-sit [g] | AP | | 0.27±0.07 | 0.26±0.06 | 0.389 |
|  | V | | 0.12±0.06 | 0.12±0.05 | 0.133 |
| Duration pitch stand -to-sit [s] | | | 1.14±0.45 | 0.95±0.30 | 0.001 * |
| Range pitch stand -to-sit [deg/s] | | | 156.20±54.49 | 150.56±37.51 | 0.669 |
| Jerk pitch stand -to-sit [deg/s^2] | | | 123.20±65.91 | 143.56±69.56 | 0.114 |
| Range stand -to-sit [g] | | ML | 0.36±0.19 | 0.29±0.12 | 0.183 |
|  | | ROLL | 40.62±19.55 | 40.28±22.11 | 0.196 |
|  | | YAW | 58.11±31.40 | 55.19±32.92 | 0.309 |

*indicates those measures that significantly differed in the two groups, after correcting for multiple comparisons (i.e., using a threshold of p=0.003). All entries are adjusted for age and gender.

| **Table 2d:** TUG signal derived measures during the two turns in subjects with and without mobility disability | | | | |
| --- | --- | --- | --- | --- |
| **Measures** | | **mobility disability** | **No mobility disability** | **P-value** |
| **Turns: Turn 1** (middle of TUG) | | | | |
| Amplitude-yaw [deg/s] | | 126.54±34.25 | 160.90±36.51 | <0.0001 * |
| Turn duration [s] | | 2.49±0.77 | 2.07±0.51 | <0.0001 * |
| Number of steps | V | 5.21±2.29 | 4.11±1.50 | <0.0001 * |
| Range [g] | V | 0.51±0.19 | 0.67±0.28 | <0.0001 * |
|  | AP | 0.36±0.13 | 0.41±0.12 | <0.0001 * |
|  | ML | 0.51±0.13 | 0.65±0.22 | <0.0001 * |
|  | YAW | 116.76±30.56 | 148.45±33.77 | <0.0001 * |
|  | PITCH | 58.34±23.53 | 65.57±23.45 | 0.009 * |
|  | ROLL | 51.71±18.67 | 63.26±25.98 | <0.0001 * |
| **Turn 2** (end of TUG) | | | | |
| Amplitude-yaw [deg/s] | | 126.00±39.81 | 161.00±38.31 | <0.0001 * |
| Turn duration [s] | | 2.33±0.75 | 2.01±0.55 | <0.0001 * |
| Number of steps | V | 3.61±1.62 | 3.36±1.54 | 0.006 * |
| Range [g] | V | 0.62±0.28 | 0.78±0.28 | <0.0001 * |
|  | AP | 0.55±0.21 | 0.63±0.16 | 0.001 * |
|  | ML | 0.58±0.18 | 0.69±0.22 | <0.0001 * |
|  | YAW | 116.63±35.76 | 149.10±34.60 | <0.0001 * |
|  | PITCH | 80.61±40.42 | 83.08±24.83 | 0.139 |
|  | ROLL | 58.34±23.08 | 68.90±24.12 | 0.001 * |

*indicates those measures that significantly differed in the two groups, after correcting for multiple comparisons (i.e., using a threshold of p=0.009). All entries are adjusted for age and gender.
